# A respiratory‐gated micro‐CT comparison of respiratory patterns in free‐breathing and mechanically ventilated rats

**DOI:** 10.14814/phy2.13074

**Published:** 2017-01-23

**Authors:** Nancy L. Ford, Lynda McCaig, Andrew Jeklin, James F. Lewis, Ruud A. W. Veldhuizen, David W. Holdsworth, Maria Drangova

**Affiliations:** ^1^ Department of Oral Biological and Medical SciencesThe University of British ColumbiaVancouverBritish ColumbiaCanada; ^2^ Department of Physics and AstronomyThe University of British ColumbiaVancouverBritish ColumbiaCanada; ^3^ Lawson Health Research InstituteLondonOntarioCanada; ^4^ Departments of Physiology and PharmacologyUniversity of Western OntarioLondonOntarioCanada; ^5^ Imaging Research LaboratoriesRobarts Research InstituteLondonOntarioCanada; ^6^ Medical BiophysicsUniversity of Western OntarioLondonOntarioCanada; ^7^ Medical ImagingUniversity of Western OntarioLondonOntarioCanada

**Keywords:** Free‐breathing, lung physiology, mechanically ventilated, micro‐computed tomography, respiratory‐gated

## Abstract

In this study, we aim to quantify the differences in lung metrics measured in free‐breathing and mechanically ventilated rodents using respiratory‐gated micro‐computed tomography. Healthy male Sprague‐Dawley rats were anesthetized with ketamine/xylazine and scanned with a retrospective respiratory gating protocol on a GE Locus Ultra micro‐CT scanner. Each animal was scanned while free‐breathing, then intubated and mechanically ventilated (MV) and rescanned with a standard ventilation protocol (56 bpm, 8 mL/kg and PEEP of 5 cm H_2_O) and again with a ventilation protocol that approximates the free‐breathing parameters (88 bpm, 2.14 mL/kg and PEEP of 2.5 cm H_2_O). Images were reconstructed representing inspiration and end expiration with 0.15 mm voxel spacing. Image‐based measurements of the lung lengths, airway diameters, lung volume, and air content were compared and used to calculate the functional residual capacity (FRC) and tidal volume. Images acquired during MV appeared darker in the airspaces and the airways appeared larger. Image‐based measurements showed an increase in lung volume and air content during standard MV, for both respiratory phases, compared with matched MV and free‐breathing. Comparisons of the functional metrics showed an increase in FRC for mechanically ventilated rats, but only the standard MV exhibited a significantly higher tidal volume than free‐breathing or matched MV. Although standard mechanical ventilation protocols may be useful in promoting consistent respiratory patterns, the amount of air in the lungs is higher than in free‐breathing animals. Matching the respiratory patterns with the free‐breathing case allowed similar lung morphology and physiology measurements while reducing the variability in the measurements.

## Introduction

Respiratory diseases are often diagnosed clinically using computed tomography (CT) due to the excellent inherent contrast between air and tissue obtained with short scan times. Preclinical micro‐CT has been developed to obtain similar images in rodent models of respiratory diseases, including emphysema (Artaechevarria et al. 2011; De Langhe et al. 2012; Ford et al. 2009; Froese et al. 2007; Kobayashi et al. 2013), asthma (Lederlin et al. 2012, 2010), and cancer (Cavanaugh et al. 2004; Cody et al. 2005; Fushiki et al. 2009; Namati et al. 2010; Rodt et al. 2012), with quantitative measurements possible to characterize the disease model (Artaechevarria et al. 2009; Ford et al. 2007a). To obtain motion‐artifact‐free images of the lung in rodents, and to characterize lung function, respiratory‐gated imaging must be employed. Different gating techniques have been proposed, including prospective techniques that synchronize image acquisition with the respiratory cycle (Badea et al. 2004; Cavanaugh et al. 2004; Cody et al. 2005; Ford et al. 2005; Walters et al. 2004) and retrospective techniques that reconstruct only those projection images that are in phase (Ford et al. 2014, 2007b; Hu et al. 2004; Sonke et al. 2005).

To achieve respiratory‐gated imaging, many researchers have used mechanical ventilation (MV) to control the respiratory cycle (Badea et al. 2004; Cavanaugh et al. 2004; Cody et al. 2005; Guerrero et al. 2006; Walters et al. 2004). The use of mechanical ventilation ensures that the respiratory pattern is stable, with each breath being nearly identical throughout the scan. Our previous studies have described respiratory‐gated imaging of free‐breathing animals (Ford et al. 2014, 2005, 2007b). Allowing the animals to breathe freely ensures that the images obtained are representative of the natural state, providing information about the structure and function of the lungs noninvasively, although there may be some variability in the respiratory pattern from one breath to the next. Our free‐breathing approach enables repeated imaging without concerns of damage to the airways as a result of intubation (Brown et al. 1999) or ventilator‐induced injuries ranging from inflammation (Li et al. 2014; Schreiber et al. 2006) to overdistension or collapse of airways (Bailey et al. 2008; Vazquez de Anda et al. 2001). Mechanical ventilation can also affect the measurements of lung structure and function (Dreyfuss and Saumon 1993), including increased lung volumes, air content, and airway diameters which may lead to erroneous conclusions about the functional state of the lung in disease models. These functional and structural alterations are strongly related to the ventilation protocol, including the respiratory rate, tidal volume, and end expiratory pressure settings used.

For micro‐CT imaging studies of rodent models of disease, some of the symptoms under investigation may be masked by overinflated airways and lung tissues resulting from mechanical ventilation. Emphysema presents as air trapping, which is identified in micro‐CT images as increased lung volume (De Langhe et al. 2012) or decreased CT number measured in the lung (Ford et al. 2009; Froese et al. 2007). Overinflating the lungs during mechanical ventilation would introduce additional air into the lungs, masking the symptoms of the disease under investigation. Conversely, fibrosis would be identified on micro‐CT images as reduced lung volumes (De Langhe et al. 2012); overinflating the lungs in this case would offset the clinical symptoms, which may limit the utility of micro‐CT imaging to more severe stages of fibrosis. To ensure that micro‐CT images are useful for monitoring the progression of respiratory diseases, including early‐stage disease, or the response to therapeutic interventions in rodent models, the images must accurately represent the disease status, with no undue influence on the measurements resulting from the image acquisition and respiratory gating methodology. Since many studies are using mechanical ventilation to control the animals’ respiration and synchronize the respiratory pattern with the micro‐CT image acquisition, understanding the impact of the ventilator parameters (respiratory rate, tidal volume) on the image‐based measurements of lung volume, air content, and respiratory function will enable researchers to ensure that appropriate ventilation strategies are employed and important markers of respiratory disease are not obscured by the measurement techniques.

The goals of this study were to identify and quantify differences in the lung morphology and physiology measurements obtained from respiratory‐gated micro‐CT images in rodents that were free‐breathing and under mechanical ventilation. In addition, we developed a mechanical ventilation protocol to match the free‐breathing conditions; in the matched MV protocol, the tidal volume and respiratory rate was set to average values for free‐breathing, age‐matched rats. We validated the measurements of lung structure and function obtained from the micro‐CT images. In this study, we performed respiratory‐gated micro‐CT scans on healthy rats using all three ventilation protocols (free‐breathing, standard MV, and matched MV) and compared the image‐based measurements of lung structure and function.

## Methods

### Animal model

Healthy male Sprague‐Dawley rats (*n* = 5, mean mass = 389.4 ± 9.4 g) were housed in a pathogen‐free vivarium under standard light–dark cycle and with free access to food and water. All animal procedures were approved by the Animal Use Subcommittee of the University of Western Ontario and performed by a trained animal care technician.

The rats were anesthetized for a single imaging session, where images were acquired using three different respiratory patterns, using an intraperitoneal injection of ketamine (75 mg/kg) and xylazine (5 mg/kg). The animals remained anesthetized throughout the approximately 75‐min imaging session. Micro‐CT images were obtained while free‐breathing, then under two different mechanical ventilation protocols. Following the free‐breathing scans, each animal underwent catheter placement in the jugular and carotid vessels to enable intravenous administration of additional anesthetic (0.2–1.5 mg/kg/min Propofol) as needed and to monitor the blood pressure and heart rate. Then the animal was endotracheally intubated with a 14‐gauge catheter, administered pancuronium bromide (0.1 mg/kg) to inhibit spontaneous respiration, and placed onto a mechanical ventilator (Flexivent, Scireq, Montreal, Que., Canada). Initially, the ventilator was connected to 100% O_2_ until the animal was stabilized, then ventilated with room air (21% O_2_) for the remainder of the scanning session. In the standard ventilation protocol, the respiration rate was 56 breaths per minute (bpm) at 8.0 mL/kg tidal volume and a positive end expiratory pressure (PEEP) of 5 cm H_2_O (Bailey et al. 2008). In a second protocol, we attempted to match the ventilator settings to the average free‐breathing respiratory rate and tidal volume that we obtained from similar animals in another study (Ford et al. 2014). In this matched protocol, the respiratory rate was 88 bpm with a tidal volume of 2.14 mL/kg and a PEEP of 2.5 cm H_2_O.

### Micro‐CT scanning

Micro‐CT imaging was performed on a high‐speed scanner (Locus Ultra, General Electric HealthCare, London, Ontario, Canada). The scanning protocol operates at 80 kVp and 50 mA, and obtained 4160 projection images in 10 consecutive gantry rotations in a total scan time of 50 sec. The entrance dose in air at the scanner isocenter has been reported previously for this protocol as 0.28 Gy (Ford et al. 2007b), which has been found to have no adverse effects on rodent lung (Detombe et al. 2013). Images were obtained first with the animal free‐breathing. Animals were then placed onto mechanical ventilation and reimaged under the standard and matched protocols described above. In order to ensure that the respiration was consistent, we waited a few minutes after changing the ventilation strategy to avoid imaging during a transitional period. We also performed the matched ventilation before and after the standard protocol to randomize the respiration and avoid any residual effects, such as lung overinflation, from the previous ventilation protocol. Multiple scans (up to 4) were performed under each ventilation strategy to ensure that no residual effects were seen. We recorded the positive inspiratory pressure (PIP) and blood pressure at the beginning and end of each ventilation protocol to identify any changes. Arterial blood gases were assessed immediately after the image acquisition for the mechanically ventilated protocols to ensure that the animal was stable and appropriately oxygenated.

Throughout the imaging session, the respiratory signal of the animal was measured using a rodent physiological monitoring device (Biovet, m2m Imaging Corp., Cleveland, OH). The rat was positioned prone on the scanning bed with a pneumatic pillow inserted between the abdomen and the table covering the diaphragm. The diaphragm motion caused a measurable change in the pressure inside the pillow, which was recorded and displayed on a laptop throughout the scan. A signal from the micro‐CT scanner indicating when the X‐rays turned on was also recorded to establish a common time frame between the image acquisition and the respiratory waveform.

### Retrospective respiratory gating and image reconstruction

To visualize the lungs at different points in the respiratory cycle, we employed a custom‐made respiratory‐sorting algorithm which used the recorded signals for respiration and X‐ray signal, and for each projection image, the algorithm identified at what point in the cycle it was acquired (Ford et al. 2014, 2007b). All projections that occurred in the desired portion of the respiratory cycle were then used for the gated reconstruction. Duplicate projections, meaning projections that were in the same respiratory phase acquired at the same angular position but in subsequent gantry rotations, were discarded. Furthermore, there may be some angular positions with no projections obtained in the desired respiratory phase, which could lead to missing view artifacts in the reconstructed images. For this study, we reconstructed images during end expiration and at peak inspiration, which are defined as the lower 20% and upper 20% of the respiratory traces, respectively. The reconstruction was done with a modified Feldkamp cone‐beam algorithm (Feldkamp et al. 1984) to produce volumetric images with a reconstructed voxel spacing of 0.15 mm. Images were rescaled into Hounsfield units to ensure that the voxel values representing air in the trachea was −1000 HU.

### Image‐based analysis

Micro‐CT images were analyzed using MicroView Analysis + (v2.2, General Electric Healthcare, London, Ontario, Canada). For each animal, all 3D micro‐CT images were registered by selecting analogous bony landmarks in the images and applying a rigid‐body transform, and reoriented to align the major airways with the image axes. A region of interest was identified for each animal that would completely enclose the entire lung field in both respiratory phases. To segment the lungs, a seeded region‐growing algorithm was used to select all connected voxels with a gray‐scale value below the threshold value. Threshold values were calculated separately for each respiratory pattern using the method described by Otsu (1979) and applied to all images within the group. To segment the lungs, we used −262 HU for free‐breathing, −382 HU for standard ventilation, and −343 HU for matched ventilation. To segment the major airways, the threshold was set at −900 HU for all images. Similar to our previous work (Ford et al. 2007a), measurements were obtained of the lung volume and lung CT density and the major airway volume and airway CT density at both peak inspiration and end expiration. Using these values, we calculated the functional residual capacity (eq. 1) and the tidal volume (eq 2).

(1) $$\text{FRC}={\text{Volume}}_{\mathrm{expiration}}\times \frac{\text{Mean Lung}\;{\text{Density}}_{\mathrm{expiration}}}{\text{Air Density}}$$

(2) $$V_{t}={\text{Volume}}_{\mathrm{inspiration}}\times \frac{\text{Mean Lung}\;{\text{Density}}_{\mathrm{inspiration}}}{\text{Air Density}}-\text{FRC}$$

Each 3D image was further processed to produce a 2D minimum intensity projection image representing the coronal view. The minimum intensity projection displays the lowest values within the region of interest, thereby highlighting the air‐filled regions of the rat. We measured the length of the right and left lungs from the apex to lower tip, the diameter of the trachea and the diameter of the left and right bronchus. The measurements of the airway diameters were obtained at a distance of 3 mm from the carina, perpendicular to the airway wall.

### Analysis of the respiratory traces

Each respiratory trace was analyzed using custom‐built scripts written in Matlab (R2012a, Mathworks, Natick MA). The respiratory trace was cropped to include only the breaths that occurred while the X‐rays were on, and smoothed with a 10‐point moving average to reduce noise. The local maxima and minima were identified and the local baseline was calculated by averaging the values from the period between adjacent breaths. A local threshold was defined as three standard deviations above the baseline value, to a maximum of 150 units, and the points on the respiratory trace that intersected the threshold were identified as the end of exhalation and the beginning of inspiration. If the script was unable to identify a point, the user was prompted to confirm or correct the point in question. Once all of the points had been located on the trace, the script performed calculations to measure the respiratory rate, average time between breaths, average time for inhalation, average time for exhalation, average breath length, and the average time spent in end expiration. The script also calculated the breath amplitude, as the average distance between the local maximum and baseline values, and the total area under the curve represented by each breath.

### Statistical methods

Statistical analysis was performed in Prism (version 6.0h, GraphPad Software, Inc., La Jolla, CA). Descriptive statistics were performed to calculate the group mean and standard deviations for each respiratory pattern and respiratory phase. Comparisons between the respiratory patterns were performed using ANOVA with Sidak's multiple comparison post hoc testing with a 95% confidence level (*P* < 0.05).

## Results

The physiological metrics measured during mechanical ventilation, including the blood pressure, PIP and the arterial blood gases, are shown in Table 1. No significant differences were observed between the matched MV protocols obtained before the standard MV and after. For all of the tabulated values, the measurements during the matched MV protocol were significantly different from those obtained during the standard MV protocol. Specifically, the oxygenation of the blood was reduced in the matched MV case, which may be due to the lower tidal volume used (2.14 mL/kg for matched MV vs. 8.0 mL/kg for standard MV). A reduction in the blood oxygen levels has been noted previously under mechanical ventilation in Sprague‐Dawley rats, with improved oxygenation being achieved with 100% O_2_ (Bailey et al. 2008). For future studies, this discrepancy may be overcome by ventilating with higher concentrations of O_2_ instead of using room air as we did in this study.

**Table 1:** Physiological and arterial blood gas measurements obtained during mechanical ventilation

	Matched MV	Standard MV	Matched MV	*P*‐value (matched‐standard)	*P*‐value (standard‐matched)	*P*‐value (matched‐matched)
Blood Pressure (mmHg)	59.6 ± 9.0	75.9 ± 11.4	57.1 ± 7.6	0.0020	0.0009	0.7383
PIP (cmH_2_O)	7.4 ± 0.6	11.4 ± 0.4	6.9 ± 0.5	0.0003	0.0001	0.6614
PaO_2_ (mmHg)	44.8 ± 7.3	85.8 ± 15.3	46.3 ± 4.7	0.0010	0.0013	0.9911
PaCO_2_ (mmHg)	51.4 ± 8.7	29.8 ± 4.6	46.2 ± 3.6	0.0144	0.0484	0.7032
%O_2_ Saturation	72.8 ± 9.0	95.8 ± 3.2	78.0 ± 7.8	0.0012	0.0048	0.4048
pH	7.28 ± 0.3	7.4 ± 0.04	7.2 ± 0.04	<0.0001	0.5489	<0.0001

The blood pressure and PIP are averaged from before and after the micro‐CT scans, and the arterial blood gases were obtained after the scans. Only four rats received the matched MV protocol before and after the standard MV and so the additional rat was excluded from this table. Tabulated values are reported as mean ± standard deviation. MV, mechanically ventilated; PIP, positive inspiratory pressure.

In four of the rats, we performed imaging under free‐breathing, matched MV, standard MV, and matched MV to ensure that no residual effects of the different ventilation strategies were observed. Preliminary analysis of the image‐based measurements showed that there were no significant differences in the images acquired under the matched MV before or after the standard MV. Since one rat did not receive the matched MV immediately following free‐breathing, we excluded the first set of matched MV scans from the other rats for consistency. We also acquired uneven numbers of scans for each rat and respiratory pattern, ranging from 2 to 4, to ensure that we were able to get at least one good scan. Some of the acquired scans had to be excluded due to technical reasons; either the lungs were not centered in the field of view, resulting in the tip of the lungs being cut off in some of the images, or the reconstructed images had artifacts due to missing projection views resulting from the retrospective sorting algorithm. If a few views are missing, the images will still have acceptable image quality, but if the missing views are adjacent to each other, streak artifacts can affect the images by placing streaks across the field of view that affect the image segmentation. To ensure a more equal comparison, we selected one scan per ventilation protocol for statistical analysis. The selection process looked at the technical reasons outlined above, and at the recorded respiratory traces to identify the most uniform respiratory patterns throughout the scan. If more than one image was deemed acceptable, we chose the last image to eliminate any residual effects from the previous ventilation scheme.

**Figure 1:**
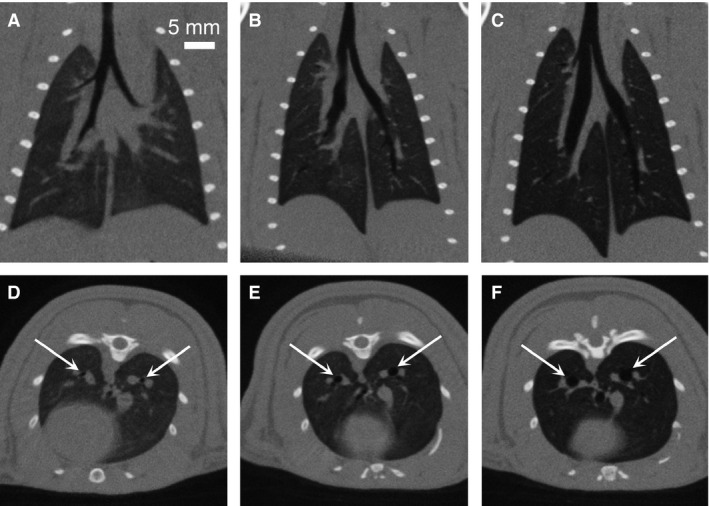
Micro‐CT images obtained during end expiration and reconstructed with 0.15 mm isotropic voxel spacing. Coronal views of a single rat while (A) free‐breathing, (B) under the matched MV protocol (88 bpm, tidal volume = 2.14 mL/kg), and (C) under standard MV (56 bpm, 8.0 mL/kg). Axial views from the same images while (D) free‐breathing, (E) matched MV, and (F) standard MV. Notice the lung parenchyma darkening and airway diameters increasing (white arrows) from the free‐breathing case (D) to the standard MV case (F) indicating more air inside the lungs and airways while under mechanical ventilation. CT, computed tomography; MV, mechanically ventilated.

**Figure 2:**
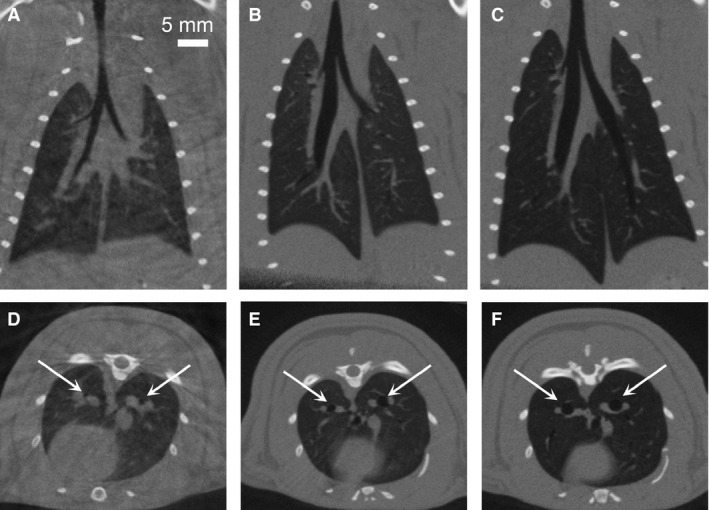
Micro‐CT images obtained during inspiration and reconstructed with 0.15 mm isotropic voxel spacing. Coronal views of the same rat as shown in Figure 1, while (A) free‐breathing, (B) under the matched MV protocol (88 bpm, tidal volume = 2.14 mL/kg), and (C) under standard MV (56 bpm, 8.0 mL/kg). Axial views from the same images while (D) free‐breathing, (E) matched MV, and (F) standard MV. Notice the lung parenchyma darkening and airway diameters increasing (white arrows) from the free‐breathing case (D) to the standard MV case (F) indicating more air inside the lungs and airways while under mechanical ventilation. In addition, the standard MV images show a marked increase in the lung volume over the other two images. CT, computed tomography; MV, mechanically ventilated.

**Figure 3:**
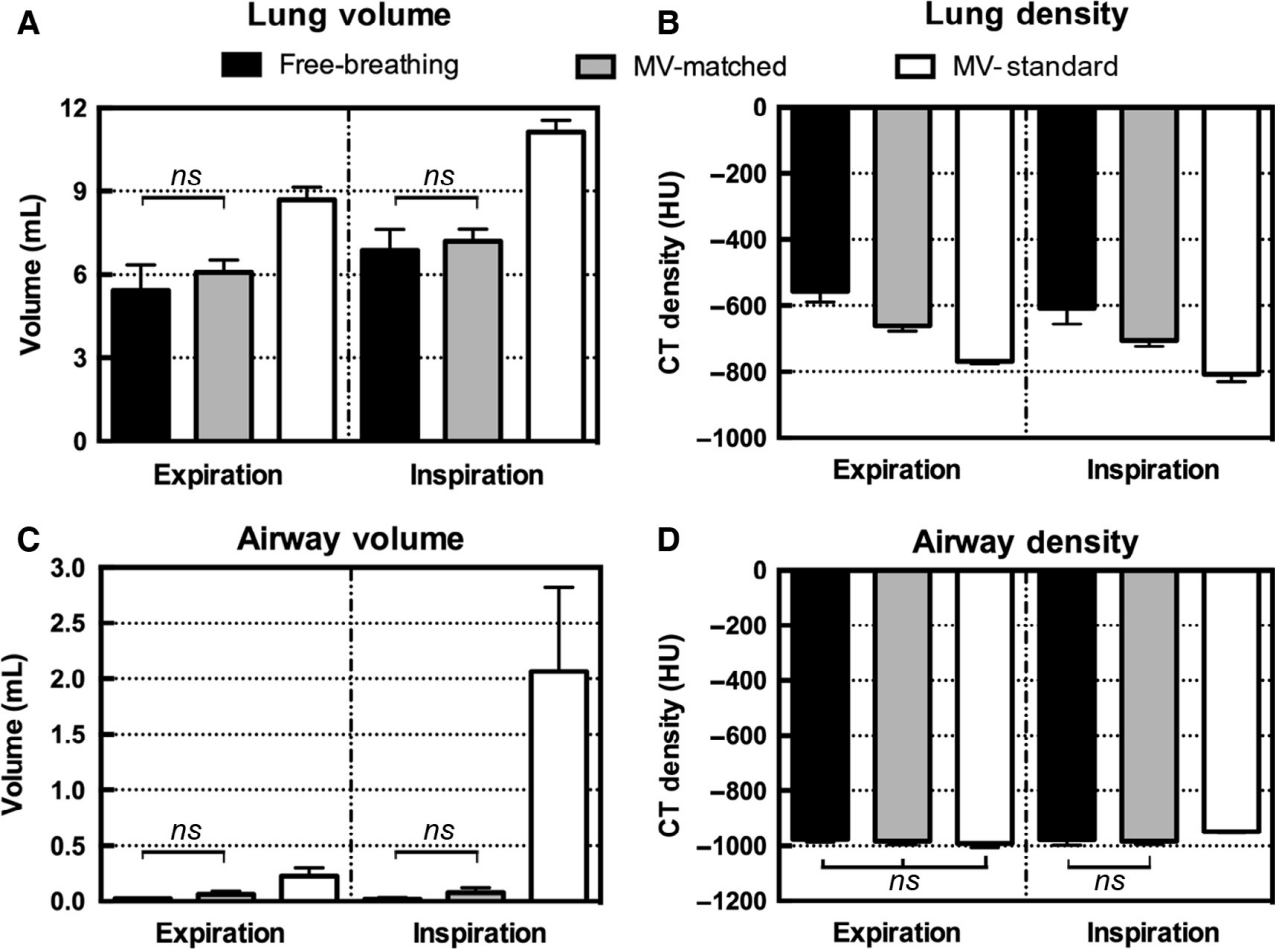
Volumetric measurements averaged for five rats obtained from the 3D micro‐CT images. Bar graphs show the mean and standard deviations for (A) lung volume (mL), (B) mean lung density (HU), (C) major airway volume (mL), and (D) mean airway density (HU). Differences that are not significant are marked on the graphs, and all other pairwise comparisons were significant (*P* < 0.05). CT, computed tomography.

Figure 1 shows the axial and coronal slices through a single rat while free‐breathing, matched MV, and standard MV during end expiration, while Figure 2 shows corresponding views during inspiration. The image‐based measurements were averaged over the five rats separately for the inspiration and end expiration images. The 3D volumetric measurements of lung volume, mean lung density, airway volume, and airway density are shown in Figure 3. The statistical analysis shows no difference between the free‐breathing and matched MV for the lung volume and the major airway volume in both respiratory phases. The standard MV was significantly different from the free‐breathing and the matched MV measurements. For the mean lung density, the measured values, which are an indicator of air content in the lungs showed an increase in air present in both respiratory phases for the matched MV and the standard MV. There were no significant differences in the mean airway densities during end expiration, but a significant difference for the standard MV during inspiration.

**Figure 4:**
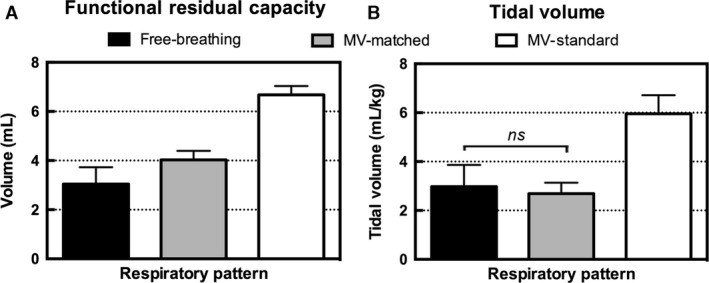
Functional metrics for five rats calculated by Equation (1), (2) using the data presented in Figure 3. Bar graphs show the mean and standard deviations for (A) functional residual capacity (mL) and (B) tidal volume (mL/kg). Differences that are not significant are marked on the graphs, and all other pairwise comparisons were significant (*P* < 0.05). CT, computed tomography.

Functional residual capacity and the tidal volume are shown in Figure 4. For both of these functional metrics, the standard MV was significantly different from the other two respiratory patterns. No difference was observed in the tidal volume of the matched MV compared with the free‐breathing respiratory pattern, as expected since the ventilator tidal volume was set to match the free‐breathing case.

**Figure 5:**
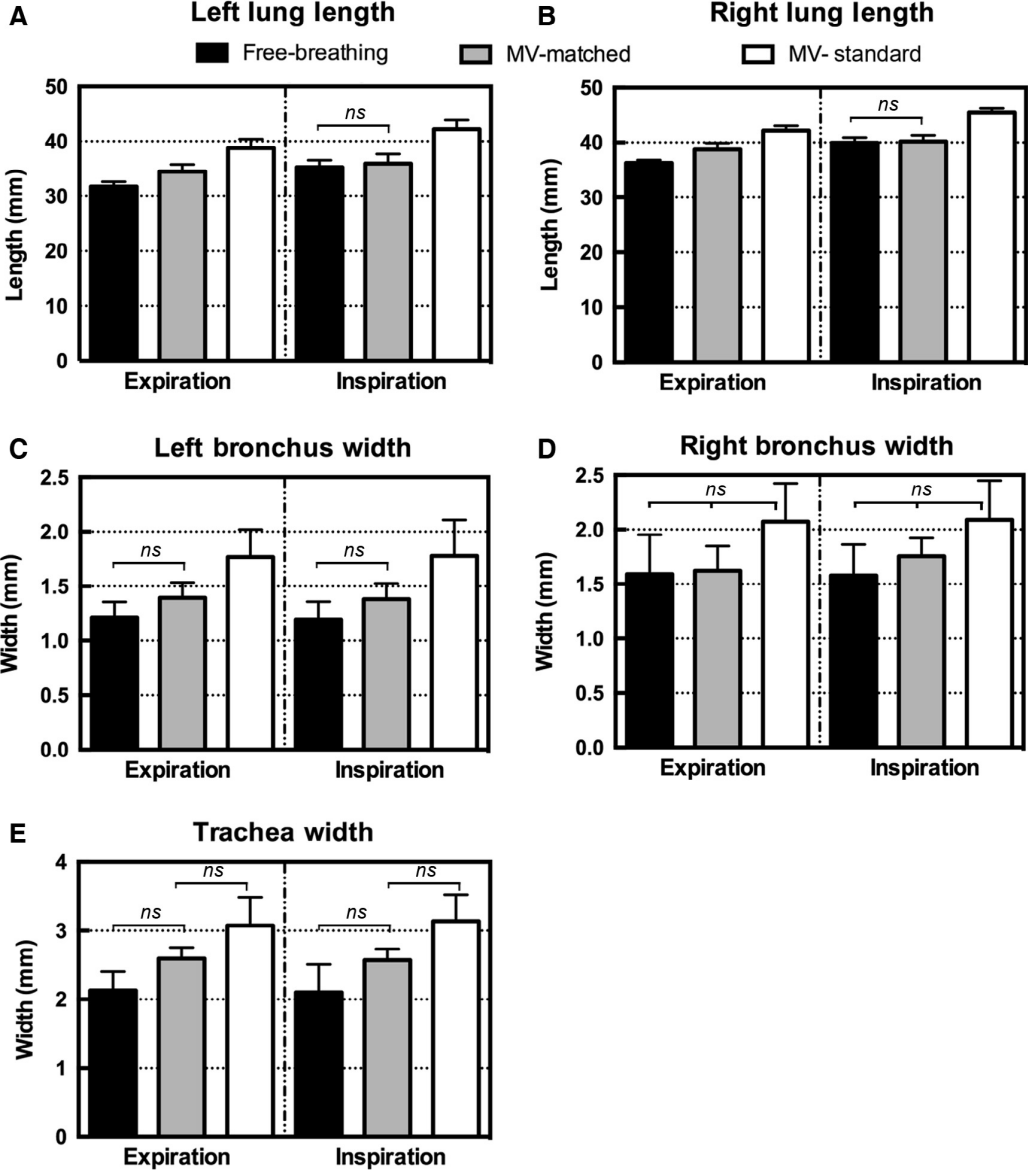
2D measurements for five rats obtained from the minimum intensity projection images. Bar graphs show the mean and standard deviations for (A) left lung length (mm) and (B) right lung length (mm) measured from apex to tip, (C) left bronchus width (mm), (D) right bronchus width (mm), and (E) trachea width (mm) obtained at 3 mm from the carina. Differences that are not significant are marked on the graphs, and all other pairwise comparisons were significant (*P* < 0.05). CT, computed tomography.

The measurements obtained from the 2D minimum intensity projection images are plotted in Figure 5. For the lung‐length measurements, there were no differences during the inspiration phase between the free‐breathing and matched MV images. For the airway diameters, no significant differences were observed in any of the measurements between free‐breathing and matched MV in either respiratory phase.

**Figure 6:**
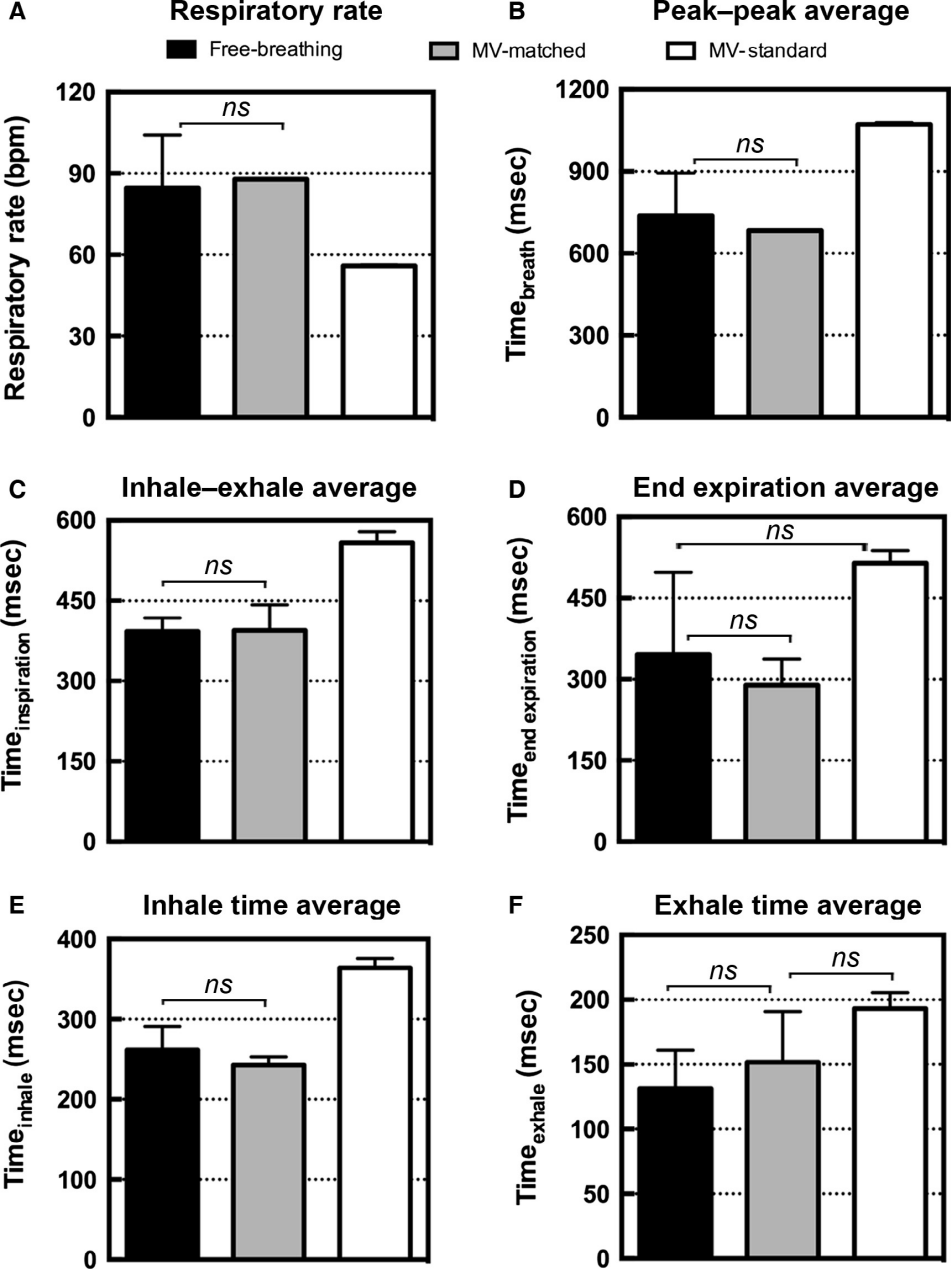
Analysis of the respiratory traces measured during the micro‐CT image acquisitions for five rats. Bar graphs show the mean and standard deviations for (A) respiratory rate (bpm), (B) average time between adjacent peaks representing a single breath (msec), (C) time from inhale to exhale representing inspiration (msec), (D) time during end expiration (msec), (E) inhalation time (msec), and (F) exhalation time (msec). Differences that are not significant are marked on the graphs, and all other pairwise comparisons were significant (*P* < 0.05). CT, computed tomography.

In Figure 6, the calculations based on the respiratory traces are shown. There are no significant differences between the free‐breathing and matched MV respiratory traces. While free‐breathing, the rats did show more breath‐to‐breath variability during each micro‐CT scan, which resulted in larger error bars for all of the measured values.

## Discussion

Comparisons of the image‐based measurements and physiological metrics for rats under standard mechanical ventilation (56 bpm, 8.0 mL/kg) compared with free‐breathing exhibited significant differences in nearly every measurement. The only measured values that were not different were the airway density during end expiration, the right bronchus width for both respiratory phases, and the average time spent in end expiration. All of the lung dimensions and volumes were increased compared to the free‐breathing case, and the air content, estimated by the CT densities, was reduced which indicates an increase in the volume of air in the lungs during the micro‐CT images at both respiratory phases. In addition, the tidal volume and functional residual capacity were both significantly increased, which shows more air was brought into the lungs per breath and more air remained after exhalation for the standard MV ventilation protocol. From these results, it is clear that the standard MV ventilation protocol overinflates the lungs of rodents leading to erroneous measurements of the structures within the lungs and the lung function.

Compared to the free‐breathing measurements, the respiratory rate was significantly lower and the tidal volume was increased for standard MV indicating that the ventilator settings were not a suitable match to the free‐breathing case. In our experience, free‐breathing animals breathe more rapidly and take shallower breaths than the standard mechanical ventilation settings. We developed a matched MV ventilation protocol to ensure that the tidal volume and respiratory rates more closely approximated the free‐breathing case. Comparing the respiratory traces of the free‐breathing and matched MV protocols, there were no significant differences in the measured respiratory patterns, although the variability in the free‐breathing traces was slightly higher due to minor differences from one breath to the next. There were no significant differences in the image‐based measurements between the free‐breathing and matched MV, except for the lung density and lung lengths during both respiratory phases and the functional residual capacity. In the matched MV case, the animals appear to have slightly more air in the lungs, leading to slightly increased volumes, densities, and FRC, but the difference is not as large as for the standard MV protocol.

This study represents the first time that image‐based lung morphology and lung function measurements have been reported in the same animals while free‐breathing and under MV. Our results show that the measurements obtained are dependent upon the ventilation strategy employed during the micro‐CT imaging session. The differences in the ventilation strategy may account for some of the discrepancies of the reported lung metrics published in the literature. We have previously measured tidal volumes in free‐breathing mice of 0.09 mL (Ford et al. 2007a), which is roughly one‐half of the typical ventilator setting of 0.18 mL. In this study, we have measured tidal volumes of 1.5 mL in free‐breathing rats compared with 2.3 mL for MV with the standard protocol. FRC values in the literature for ventilated mice, reported by Mitzner et al. as 0.35 mL for C3H/HeJ and 0.25 mL for C57/Bl6 mice, are increased compared to our previous publication reporting 0.16 mL in free‐breathing C57/Bl6 mice (Ford et al. 2007a). This study also shows a reduction by one‐half comparing the free‐breathing FRC of 3.0 mL to the standard MV FRC of 6.7 mL. By developing a matched MV protocol, we have been able to bring the measured lung structure and function metrics more closely in line with those obtained in free‐breathing animals, which will serve to ensure consistency in the literature for these types of metrics.

Overinflation of rodent lungs due to the standard MV protocol may lead to erroneous conclusions about the disease model under investigation. Overinflation may force the tissues to expand beyond the natural state, which may reduce the ability of the micro‐CT scan to detect and characterize the degree of disease in models that exhibit changes in tissue elasticity and compliance (Leco et al. 2001; Martin et al. 2007). Overinflation due to MV may also induce lung injury, which manifests as inflammation (dos Santos and Slutsky 2006; Li et al. 2014; Schreiber et al. 2006; Tremblay et al. 1997) or distension/collapse of structures (Bailey et al. 2008; Vazquez de Anda et al. 2001), and can propagate the pathophysiological processes of various disease models. To ensure that the imaging procedure does not interfere with the interpretation of the measured values, or impact the well‐being of an animal that is recovered for additional experimental procedures following the micro‐CT scanning session, the animal should be maintained in a respiratory state that is more natural, either through the use of free‐breathing imaging techniques or by matching the ventilator settings to more closely represent the free‐breathing state. In this study, we have demonstrated the feasibility of image‐based measurements in healthy animals for both free‐breathing and matched MV, with excellent agreement in the measured values.

## Conclusions

In this study, we have quantified the differences in the image‐based measurements of lung structure and function due to respiratory patterns in healthy rats. We found that the standard MV (56 bpm, 8.0 mL/kg) protocol led to overinflated lungs observed in the micro‐CT images compared to the free‐breathing protocol. We also demonstrated that the image‐based measurements of lung structure and function returned to levels similar to the free‐breathing protocol when the ventilator settings more closely matched free‐breathing (88 bpm, 2.14 mL/kg).

## Conflict of Interest

None declared.

## References

[phy213074-bib-0001] Artaechevarria, X. , D. Perez‐Martin , M. Ceresa , G. de Biurrun , D. Blanco , L. M. Montuenga , et al. 2009 Airway segmentation and analysis for the study of mouse models of lung disease using micro‐CT. Phys. Med. Biol. 54:7009–7024. 19887716 10.1088/0031-9155/54/22/017

[phy213074-bib-0002] Artaechevarria, X. , D. Blanco , G. de Biurrun , M. Ceresa , D. Perez‐Martin , G. Bastarrika , et al. 2011 Evaluation of micro‐CT for emphysema assessment in mice: comparison with non‐radiological techniques. Eur. Radiol. 21:954–962. 20953986 10.1007/s00330-010-1982-5

[phy213074-bib-0003] Badea, C. , L. W. Hedlund , and G. A. Johnson . 2004 Micro‐CT with respiratory and cardiac gating. Med. Phys. 31:3324–3329. 15651615 10.1118/1.1812604 PMC1357396

[phy213074-bib-0004] Bailey, T. C. , A. A. Maruscak , E. L. Martin , A. R. Forbes , A. Petersen , L. A. McCaig , et al. 2008 The effects of long‐term conventional mechanical ventilation on the lungs of adult rats. Crit. Care Med. 36:2381–2387. 18596632 10.1097/CCM.0b013e318180b65c

[phy213074-bib-0005] Brown, R. H. , D. M. Walters , R. S. Greenberg , and W. Mitzner . 1999 A method of endotracheal intubation and pulmonary functional assessment for repeated studies in mice. J. Appl. Physiol. 87:2362–2365. 10601190 10.1152/jappl.1999.87.6.2362

[phy213074-bib-0006] Cavanaugh, D. , E. Johnson , R. E. Price , J. Kurie , E. L. Travis , and D. D. Cody . 2004 In vivo respiratory‐gated micro‐CT imaging in small‐animal oncology models. Mol. Imaging 3:55–62. 15142412 10.1162/15353500200403184

[phy213074-bib-0007] Cody, D. D. , C. L. Nelson , W. M. Bradley , M. Wislez , D. Juroske , R. E. Price , et al. 2005 Murine lung tumor measurement using respiratory‐gated micro‐computed tomography. Invest. Radiol. 40:263–269. 15829823 10.1097/01.rli.0000160070.67270.05

[phy213074-bib-0008] De Langhe, E. , G. Vande Velde , J. Hostens , U. Himmelreich , B. Nemery , F. P. Luyten , et al. 2012 Quantification of lung fibrosis and emphysema in mice using automated micro‐computed tomography. PLoS ONE 7:e43123. 22912805 10.1371/journal.pone.0043123 PMC3418271

[phy213074-bib-0009] Detombe, S. A. , J. Dunmore‐Buyze , I. E. Petrov , and M. Drangova . 2013 X‐ray dose delivered during a longitudinal micro‐CT study has no adverse effect on cardiac and pulmonary tissue in C57BL/6 mice. Acta Radiol. (Stockholm, Sweden: 1987) 54: 435–441. 23436828 10.1177/0284185113475608

[phy213074-bib-0010] Dreyfuss, D. , and G. Saumon . 1993 Role of tidal volume, FRC, and end‐inspiratory volume in the development of pulmonary edema following mechanical ventilation. Am. Rev. Respir. Dis. 148:1194–1203. 8239153 10.1164/ajrccm/148.5.1194

[phy213074-bib-0011] Feldkamp, L. A. , L. C. Davis , and J. W. Kress . 1984 Practical Cone‐Beam Algorithm. J. Opt. Soc. Am. A‐Opt. Image Sci. Vis. 1:612–619.

[phy213074-bib-0012] Ford, N. L. , H. N. Nikolov , C. J. Norley , M. M. Thornton , P. J. Foster , M. Drangova , et al. 2005 Prospective respiratory‐gated micro‐CT of free breathing rodents. Med. Phys. 32:2888–2898. 16266103 10.1118/1.2013007

[phy213074-bib-0013] Ford, N. L. , E. L. Martin , J. F. Lewis , R. A. Veldhuizen , M. Drangova , and D. W. Holdsworth . 2007a In vivo characterization of lung morphology and function in anesthetized free‐breathing mice using micro‐computed tomography. J. Appl. Physiol. 102:2046–2055. 17255374 10.1152/japplphysiol.00629.2006

[phy213074-bib-0014] Ford, N. L. , A. R. Wheatley , D. W. Holdsworth , and M. Drangova . 2007b Optimization of a retrospective technique for respiratory‐gated high speed micro‐CT of free‐breathing rodents. Phys. Med. Biol. 52:5749–5769. 17881798 10.1088/0031-9155/52/19/002

[phy213074-bib-0015] Ford, N. L. , E. L. Martin , J. F. Lewis , R. A. Veldhuizen , D. W. Holdsworth , and M. Drangova . 2009 Quantifying lung morphology with respiratory‐gated micro‐CT in a murine model of emphysema. Phys. Med. Biol. 54:2121–2130. 19287083 10.1088/0031-9155/54/7/018

[phy213074-bib-0016] Ford, N. L. , A. Jeklin , K. Yip , D. Yohan , D. W. Holdsworth , and M. Drangova . 2014 Optimization of image quality in retrospective respiratory‐gated micro‐CT for quantitative measurements of lung function in free‐breathing rats. J. Biomed. Sci. Eng. 07:157–172.

[phy213074-bib-0017] Froese, A. R. , K. Ask , R. Labiris , T. Farncombe , D. Warburton , M. D. Inman , et al. 2007 Three‐dimensional computed tomography imaging in an animal model of emphysema. Eur. Respir. J. 30:1082–1089. 17804451 10.1183/09031936.00000507

[phy213074-bib-0018] Fushiki, H. , T. Kanoh‐Azuma , M. Katoh , K. Kawabata , J. Jiang , N. Tsuchiya , et al. 2009 Quantification of mouse pulmonary cancer models by microcomputed tomography imaging. Cancer Sci. 100:1544–1549. 19459854 10.1111/j.1349-7006.2009.01199.x PMC11158256

[phy213074-bib-0019] Guerrero, T. , K. Sanders , E. Castillo , Y. Zhang , L. Bidaut , T. Pan , et al. 2006 Dynamic ventilation imaging from four‐dimensional computed tomography. Phys. Med. Biol. 51:777–791. 16467578 10.1088/0031-9155/51/4/002

[phy213074-bib-0020] Hu, J. , S. T. Haworth , R. C. Molthen , and C. A. Dawson . 2004 Dynamic small animal lung imaging via a postacquisition respiratory gating technique using micro‐cone beam computed tomography. Acad. Radiol. 11:961–970. 15350577 10.1016/j.acra.2004.05.019

[phy213074-bib-0021] Kobayashi, S. , R. Fujinawa , F. Ota , S. Kobayashi , T. Angata , M. Ueno , et al. 2013 A single dose of lipopolysaccharide into mice with emphysema mimics human chronic obstructive pulmonary disease exacerbation as assessed by micro‐computed tomography. Am. J. Respir. Cell Mol. Biol. 49:971–977. 23822858 10.1165/rcmb.2013-0074OC

[phy213074-bib-0022] Leco, K. J. , P. Waterhouse , O. H. Sanchez , K. L. Gowing , A. R. Poole , A. Wakeham , et al. 2001 Spontaneous air space enlargement in the lungs of mice lacking tissue inhibitor of metalloproteinases‐3 (TIMP‐3). J. Clin. Invest. 108:817–829. 11560951 10.1172/JCI12067 PMC200926

[phy213074-bib-0023] Lederlin, M. , A. Ozier , M. Montaudon , H. Begueret , O. Ousova , R. Marthan , et al. 2010 Airway remodeling in a mouse asthma model assessed by in‐vivo respiratory‐gated micro‐computed tomography. Eur. Radiol. 20:128–137. 19685058 10.1007/s00330-009-1541-0

[phy213074-bib-0024] Lederlin, M. , A. Ozier , G. Dournes , O. Ousova , P. O. Girodet , H. Begueret , et al. 2012 In vivo micro‐CT assessment of airway remodeling in a flexible OVA‐sensitized murine model of asthma. PLoS ONE 7:e48493. 23119036 10.1371/journal.pone.0048493 PMC3484051

[phy213074-bib-0025] Li, H. , C. Wang , J. Hu , and J. Tan . 2014 A study on circadian rhythm disorder of rat lung tissue caused by mechanical ventilation induced lung injury. Int. Immunopharmacol. 18:249–254. 24355794 10.1016/j.intimp.2013.12.001

[phy213074-bib-0026] Martin, E. L. , E. A. Truscott , T. C. Bailey , K. J. Leco , L. A. McCaig , J. F. Lewis , et al. 2007 Lung mechanics in the TIMP3 null mouse and its response to mechanical ventilation. Exp. Lung Res. 33:99–113. 17454105 10.1080/01902140701198625

[phy213074-bib-0027] Namati, E. , J. Thiesse , J. C. Sieren , A. Ross , E. A. Hoffman , and G. McLennan . 2010 Longitudinal assessment of lung cancer progression in the mouse using in vivo micro‐CT imaging. Med. Phys. 37:4793–4805. 20964199 10.1118/1.3476454 PMC2937054

[phy213074-bib-0028] Otsu, N. 1979 A threshold selection method from gray‐level histograms. IEEE Transactions on Systems, Man, and Cybernetics 9:62–66.

[phy213074-bib-0029] Rodt, T. , C. von Falck , S. Dettmer , K. Hueper , R. Halter , L. Hoy , et al. 2012 Lung tumour growth kinetics in SPC‐c‐Raf‐1‐BB transgenic mice assessed by longitudinal in‐vivo micro‐CT quantification. J. Exp. Clin. Cancer Res. 31:15. 22348342 10.1186/1756-9966-31-15 PMC3308131

[phy213074-bib-0030] dos Santos, C. C. , and A. S. Slutsky . 2006 The contribution of biophysical lung injury to the development of biotrauma. Annu. Rev. Physiol. 68:585–618. 16460285 10.1146/annurev.physiol.68.072304.113443

[phy213074-bib-0031] Schreiber, T. **,** C. Niemann **,** B. Schmidt , and W. Karzai . 2006 A novel model of selective lung ventilation to investigate the long‐term effects of ventilation‐induced lung injury. Shock 26: 50–54. 16783198 10.1097/01.shk.0000215318.99241.25

[phy213074-bib-0032] Sonke, J. J. , L. Zijp , P. Remeijer , and M. van Herk . 2005 Respiratory correlated cone beam CT. Med. Phys. 32:1176–1186. 15895601 10.1118/1.1869074

[phy213074-bib-0033] Tremblay, L. , F. Valenza , S. P. Ribeiro , J. Li , and A. S. Slutsky . 1997 Injurious ventilatory strategies increase cytokines and c‐fos m‐RNA expression in an isolated rat lung model. J. Clin. Invest. 99:944–952. 9062352 10.1172/JCI119259 PMC507902

[phy213074-bib-0034] Vazquez de Anda, G. F. . R. A. Lachmann , S. J. Verbrugge , D. Gommers , J. J. Haitsma , and B. Lachmann . 2001 Partial liquid ventilation improves lung function in ventilation‐induced lung injury. Eur. Respir. J. 18:93–99. 11510811 10.1183/09031936.01.00019901

[phy213074-bib-0035] Walters, E. B. , K. Panda , J. A. Bankson , E. Brown , and D. D. Cody . 2004 Improved method of in vivo respiratory‐gated micro‐CT imaging. Phys. Med. Biol. 49:4163–4172. 15470930 10.1088/0031-9155/49/17/023

